# Surveillance of Human Rotaviruses in Wuhan, China (2019–2022): Whole-Genome Analysis of Emerging DS-1-like G8P[8] Rotavirus

**DOI:** 10.3390/ijms241512189

**Published:** 2023-07-29

**Authors:** Xuan Zhou, Yuanhong Wang, Nan Chen, Beibei Pang, Manqing Liu, Kun Cai, Nobumichi Kobayashi

**Affiliations:** 1 Division of Microbiology, Wuhan Center for Disease Control and Prevention, Wuhan 430024, China; ice2bhi@gmail.com (X.Z.); pangbei0429@163.com (B.P.); liumq33@hotmail.com (M.L.); 2 Department of Aquatic Animal Medicine, College of Fisheries, Huazhong Agricultural University, Wuhan 430070, China; chennan@mail.hzau.edu.cn; 3 Institute of Health Inspection and Testing, Hubei Provincial Center for Disease Control and Prevention, Wuhan 430079, China; ckreal@163.com; 4 Department of Hygiene, Sapporo Medical University School of Medicine, Sapporo 060-8556, Japan; nkobayas@sapmed.ac.jp

**Keywords:** rotavirus, G8, G2P[4], genome, reassortment, COVID-19 era

## Abstract

Group A rotaviruses (RVAs) are major etiologic agents of gastroenteritis in infants and young children worldwide. To study the prevalence and genetic characteristics of RVAs, a hospital-based surveillance study was conducted in Wuhan, China from June 2019 through May 2022. The detection rates of RVAs were 19.40% (142/732) and 3.51% (8/228) in children and adults, respectively. G9P[8] was the predominant genotype, followed by G8P[8] and G3P[8]. G8P[8] emerged and was dominant in the 2021–2022 epidemic season. The genome constellation of six G8P[8] strains was assigned to G8-P[8]-I2-R2-C2-M2-A2-N2-T2-E2-H2. Phylogenetic analysis revealed that the VP7, VP4, VP2, VP3, NSP1, NSP2, NSP3, and NSP5 genes of these G8P[8] strains clustered closely with those of the G8P[8] strains in Asia and were distant from those of the P[8] and G2P[4] strains simultaneously detected in Wuhan. In contrast, the VP1, VP6, and NSP4 genes were closely related to the typical G2P[4] rotavirus, including those of G2P[4] strains simultaneously detected in Wuhan. The detection rate of RVAs decreased in the COVID-19 pandemic era. It was deduced that the G8P[8] rotaviruses that emerged in China may be reassortants, carrying the VP6, VP1, and NSP4 genes derived from the G2P[4] rotavirus in the backbone of the neighboring DS-1-like G8P[8] strains represented by CAU17L-103.

## 1. Introduction

Group A rotaviruses (RVAs) are the leading cause of severe diarrhea in children less than 5 years of age globally, being responsible for an estimated 128,500 deaths in 2016 alone [1]. More than 65% of these deaths are estimated to occur in 11 countries in Asia and Africa [2,3]. Vaccination is an essential strategy for the prevention and control of severe disease caused by rotavirus infection. Until now, a total of four vaccines have been introduced by WHO to the world [4].

According to the International Committee on the Taxonomy of Viruses (ICTV), rotaviruses are 11-segmented double-stranded RNA viruses that can be classified into nine species (from A to J) belonging to the genus *Rotavirus* in the family *Sedoreoviridae*. Rotaviruses possess six structural viral proteins (VP1–VP4, VP6, and VP7) and six non-structural proteins (NSP1–NSP5/6) [5]. Each gene is differentiated into distinct genotypes according to a predefined nucleotide sequence identity. The outer layer protein, VP7, and spike protein, VP4, of rotaviruses are neutralization antigens, determining the virus genotypes G (glycoprotein; VP7) and P (protease-sensitive; VP4). RVAs have been found in various host species, including birds and mammals. At least forty-two G types and fifty-eight P types of RVAs have been differentiated so far in the world [6]. Although more than 60 G-P combinations have been reported in human RVAs, G1P[8], G2P[4], G3P[8], G4P[8], and G9P[8] are the most common G-P combinations in the world. The predominant rotavirus strain shifted from G1P[8] to G3P[8] in 2001; G3P[8] was replaced by G9P[8] after 2012 in China [7,8]. The rare G-P combination G8P[8] emerged in the south of China in 2020–2021 [9,10].

An extended classification and the nomenclature of RVAs have been established to define genotypes for 11 segments of the whole genome by the Rotavirus Classification Working Group (RCWG). The nomenclature Gx-P[x]-Ix-Rx-Cx-Mx-Ax-Nx-Tx-Ex-Hx represents genotypes of the VP7-VP4-VP6-VP1-VP2-VP3-NSP1-NSP2-NSP3-NSP4-NSP5-encoding gene segments, respectively, with “x” indicating genotype numbers [11]. Genotyping and phylogenetic analysis of human RVA strains demonstrate their close genetic relatedness to animal RVA strains, and a comprehensive genetic analysis of all 11 genome segments revealed that the two major human genotype constellations, the Wa-like (I1-R1-C1-M1-A1-N1-T1-E1-H1) and DS-1-like (I2-R2-C2-M2-A2-N2-T2-E2-H2) genogroups, have a common origin with porcine and bovine RVA strains, respectively [12]. It has been reported that the genomic constellations of human G8 rotaviruses usually have a DS-1-like backbone (I2-R2-C2-M2-A2-N2-T2-E2-H2).

Some rotavirus strains seem to be well adapted in several species, whilst others are species-specific. Reassortment and interspecies transmission may generate novel human RVA strains [13,14]. The G8 RVA is commonly regarded as being of bovine origin [15,16]. The first human G8 rotavirus, named 69M (G8-P[10]-I2-R2-C2-M2-A2-N2-T2-E2-H2), was identified in Indonesia in 1980 [16,17]. The human G8P[8] rotavirus spread in East and Southeast Asian countries, including Thailand, Japan, and Vietnam, from 2010 through 2019 [18,19,20,21,22,23].

A constant surveillance of human rotaviruses in all age groups with acute gastroenteritis has been conducted since 2000 in Wuhan, China [24,25,26,27]. The aims of the present study were to update the data on the surveillance of human rotaviruses in Wuhan during the era of the COIVD-19 pandemic and to reveal the possible origin and molecular evolution of the emerging G8 strain based on analysis of its full genome.

## 2. Results

### 2.1. Detection of Rotaviruses and VP7 and VP4 Genotyping

A total of 960 fecal specimens were collected from June 2019 through May 2022. RVAs were detected in 19.40% (142/732) and 3.51% (8/228) of the specimens from children (under 15 years old) and adults (15–96 years old), respectively. Rotaviruses B and C were not detected. The highest detection rate was 31.1% (66/212) in the age group of 13–24 months, followed by 29.2% (21/72) in those aged 25–36 months. The detection rates in the other age groups, including those aged 0–6 months, 7–12 months, 37–48 months, 49–60 months, 6–14 years, 15–59 years, and over 60 years were 3.4–15.2% (χ^2^ = 15.27, *p* < 0.05). Briefly, the major signs and symptoms of the patients infected with rotavirus were 3–20 loose stools and 0–6 episodes of vomiting within 24 h. Most of the patients were not dehydrated, and several patients had mild to moderate dehydration. The specific clinical signs were not associated with the specific genotypes of rotaviruses in the present study. Patients infected with rotaviruses of different genotypes had severe/moderate or mild signs and symptoms.

Due to the outbreak of coronavirus disease (COVID-19), the period of the present study was briefly divided into three phases. Phase 1 (pre-COVID-19 period) was from June of 2019 to the end of 2019. Phase 2 (outbreak and lockdown period) was from the beginning of 2020 through May 2020. Phase 3 (zero-COVID-19 period) was from June 2020 to May 2022. In phase 1, the detection rates of RVAs were 12.4% (38/307) and 1.9% (3/161) from children and adults, respectively. In phase 2, only twenty-five and five fecal specimens were collected and RVAs were detected in children and adults 76% (19/25) and 20% (1/5), respectively. In phase 3, the detection rates of RVAs were 21.3% (85/400) and 6.5% (4/62) from children and adults, respectively.

A total of five G-P combinations were determined throughout the study period. Generally, G9P[8] was dominant (64.7%), followed by G8P[8] and G3P[8] (14.7% for each), G2P[4] (3.3%), and G1P[8] (2.7%) (Table 1). Of these, G9P[8] was predominant both in children (62.7%) and adults (100.0%). During the study period, G9P[8] was dominant except for the epidemic season of 2021–2022, when G8P[8] was more frequent. In the present study, the first G8 rotavirus strain was collected on 8 December 2021.

### 2.2. The Genotype Constellation of RVAs

In order to explore the possible origin of the G8P[8] rotavirus detected in Wuhan, the whole genome of simultaneous co-circulating viruses, four Wa-like G1P[8]/G9P[8] and two DS-1-like G2P[4] rotaviruses, were determined and analyzed together with the G8P[8] rotavirus (Table 2). The genome of the G8 strains was assigned to the G8-P[8]-I2-R2-C2-M2-A2-N2-T2-E2-H2 genotype, which showed the genetic constellation of the DS-1-like human rotavirus genogroup. All the G9P[8] and G1P[8] strains showed the genetic constellation of Wa-like human rotaviruses (Table 2). Among all the G8P[8] strains included in Table 2, the nucleotide sequence identities of the VP7 genes and the VP4 genes were 98.2–100%. The nucleotide identities of the VP2, VP3, VP6, NSP1, NSP3, NSP4, and NSP5 genes among these DS-1-like G8 strains were over 91.3%, whereas the VP1 and NSP2 genes showed lower identities, with a minimum value of 85.9% and 87.0%, respectively (Table 2). The 11 genes of the G8P[8] strains in the present study showed 99.4–100% nucleotide identities to those of the two G8P[8] strains detected in Guangzhou City, China (GZ-0005 and GZ-0013).

### 2.3. Phylogenetic Analysis

#### 2.3.1. Structural Protein Genes

**Figure 1:**
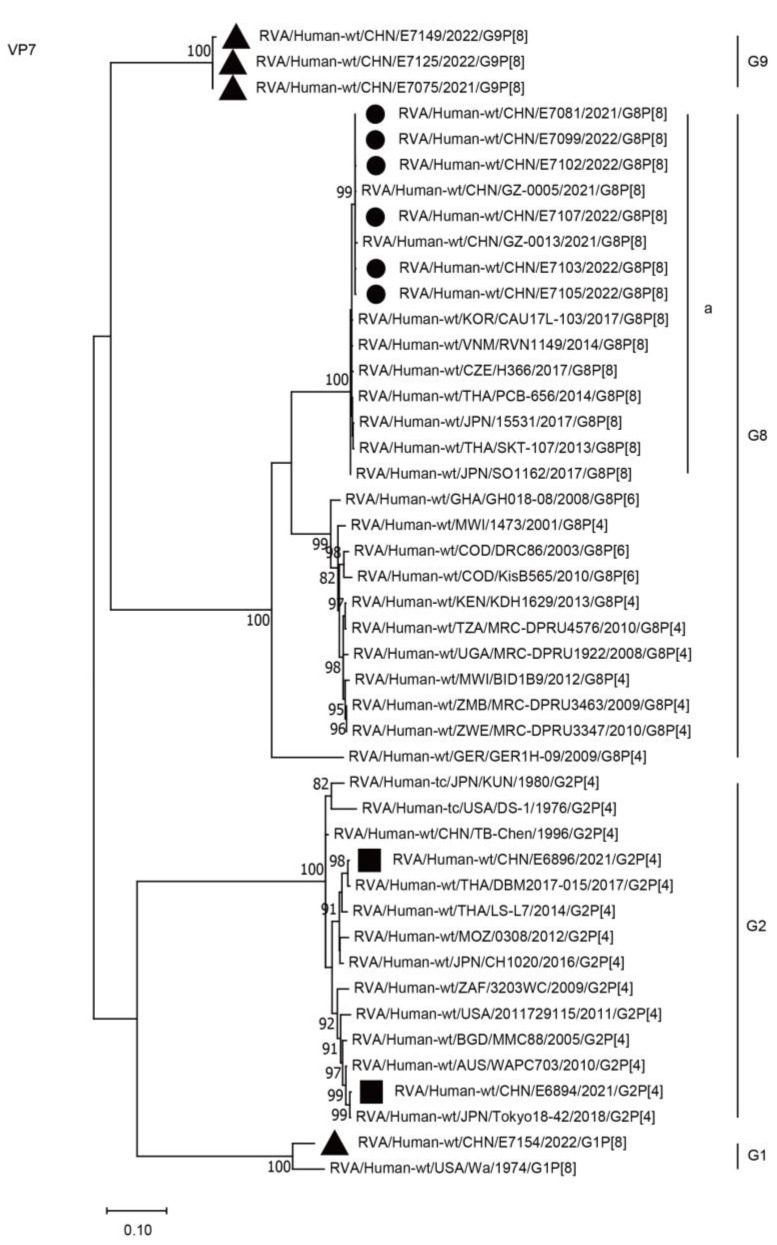
Phylogenetic dendrogram based on complete coding regions of the VP7 genes of representative RVAs. The best nucleotide substitution model was T92 + G. Bootstrap values below 80% are not shown. The G8P[8] strains are highlighted with a filled circle. The contemporaneous G2 and G1/G9 strains are highlighted with filled squares and triangles, respectively. “a” represents a lineage of G8P[8] within G8.

Phylogenetically, the VP7 genes of E7081, E7099, E7102, E7103, E7105, and E7107 were grouped into the G8 genotype and clustered together in lineage “a” with those of the strains detected in China and neighboring countries, including Guangzhou City in China (GZ-0005, GZ-0013), Japan (15531), Korea (CAU17L-103), Thailand (PCB656), and Vietnam (RVN1149), with a minimum nucleotide identity of 99.2% (Figure 1, G8a; Table 2). The VP7 genes of E6894/E6896, E7154, and E7075/E7125/E7149 were grouped into the G2, G1, and G9 genotypes, respectively (Figure 1, Table 2).

**Figure 2:**
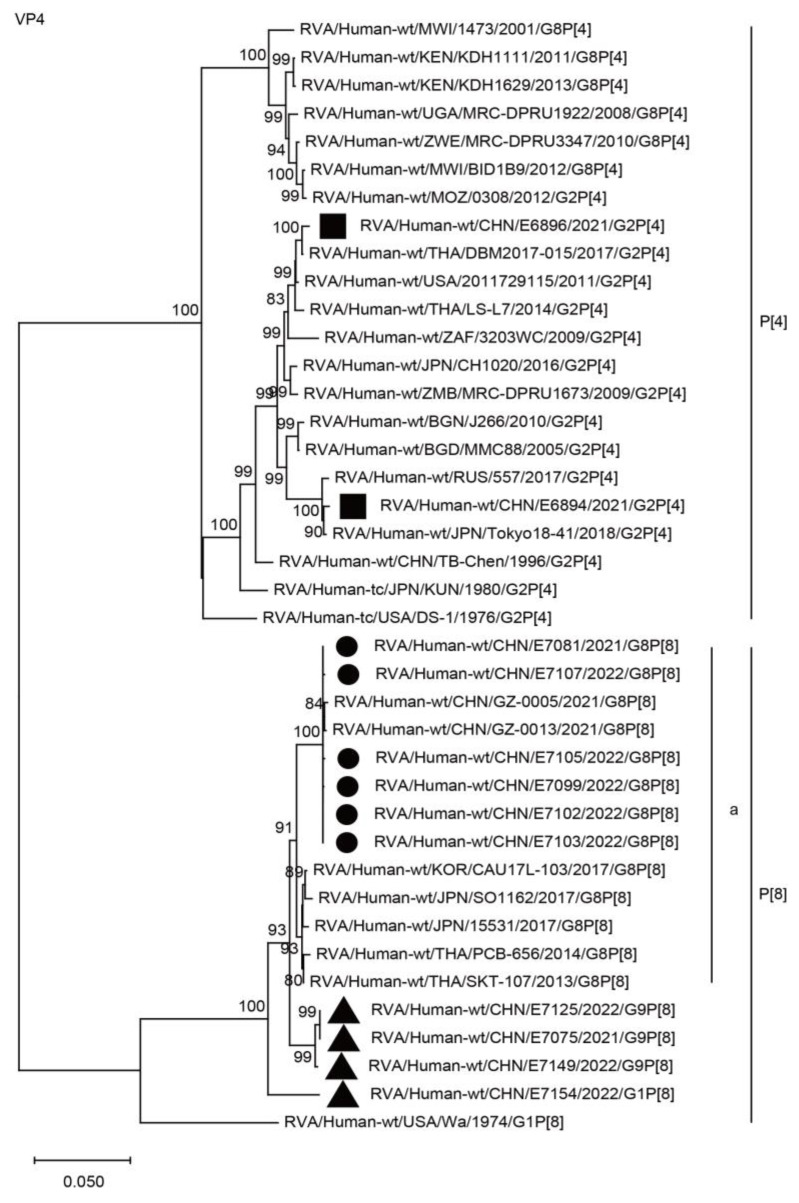
Phylogenetic dendrogram based on complete coding regions of the VP4 genes of representative RVAs. The best nucleotide substitution model was T92 + G. Bootstrap values below 80% are not shown. The G8P[8] strains are highlighted with a filled circle. The contemporaneous G2 and G1/G9 strains are highlighted with filled squares and triangles, respectively. “a” represents a lineage of G8P[8] within P[8].

The VP4 genes of the six G8 strains in the present study and four simultaneous co-circulating G1 and G9 strains were grouped into the P[8] genotype. The six G8 RVAs were closely related to those of the G8P[8] strains from China and neighboring countries, such as GZ-0005, SO1162, CAU17L-103, and PCB656, showing a minimum nucleotide identity of 98.1%, and they belonged to lineage “a” (Figure 2, P[8]a; Table 2) and were distant from the co-circulating G1P[8] and G9P[8] strains, with nucleotide identities of 95.7–97.2%. The VP4 genes of E6894 and E6896 were assigned to the P[4] genotype and were close to other G2P[4] strains found in Asia, Europe, Africa, and America during the years of 2005–2018.

**Figure 3:**
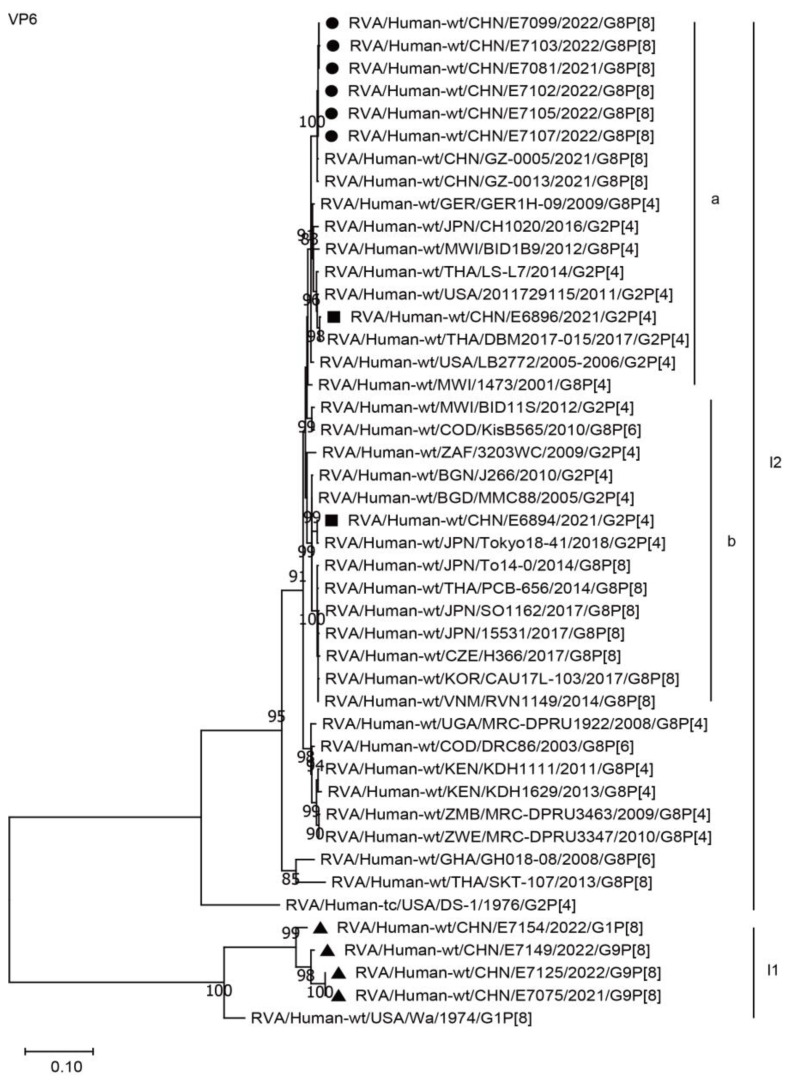
Phylogenetic dendrogram based on complete coding regions of the VP6 genes of representative RVAs. The best nucleotide substitution model was T92 + G. Bootstrap values below 80% are not shown. The G8P[8] strains are highlighted with a filled circle. The contemporaneous G2 and G1/G9 strains are highlighted with filled squares and triangles, respectively. “a” and “b” represent lineages within I2 (G8P[8] strains, lineage “a”).

The VP6 genes of the six G8 strains in the present study were assigned to the I2 genotype. These genes are closely related to two Chinese G8P[8] strains from Guangzhou City and the G8P[4] strain GER1H-09 from Germany, as well as the G2P[4] strains, such as the American strain LB2772 and the Chinese simultaneous co-circulating G2P[4] strain E6896, in the present study, with a minimum nucleotide identity of 97.7%, while remaining distant from the G8[8] strains found in Asia and Europe, with a maximum nucleotide identity of 96.6% (Figure 3, I2a and I2b; Table 2). The VP6 genes of the simultaneous co-circulating G1 and G9 strains were assigned to the I1 genotype (Figure 3).

**Figure 4:**
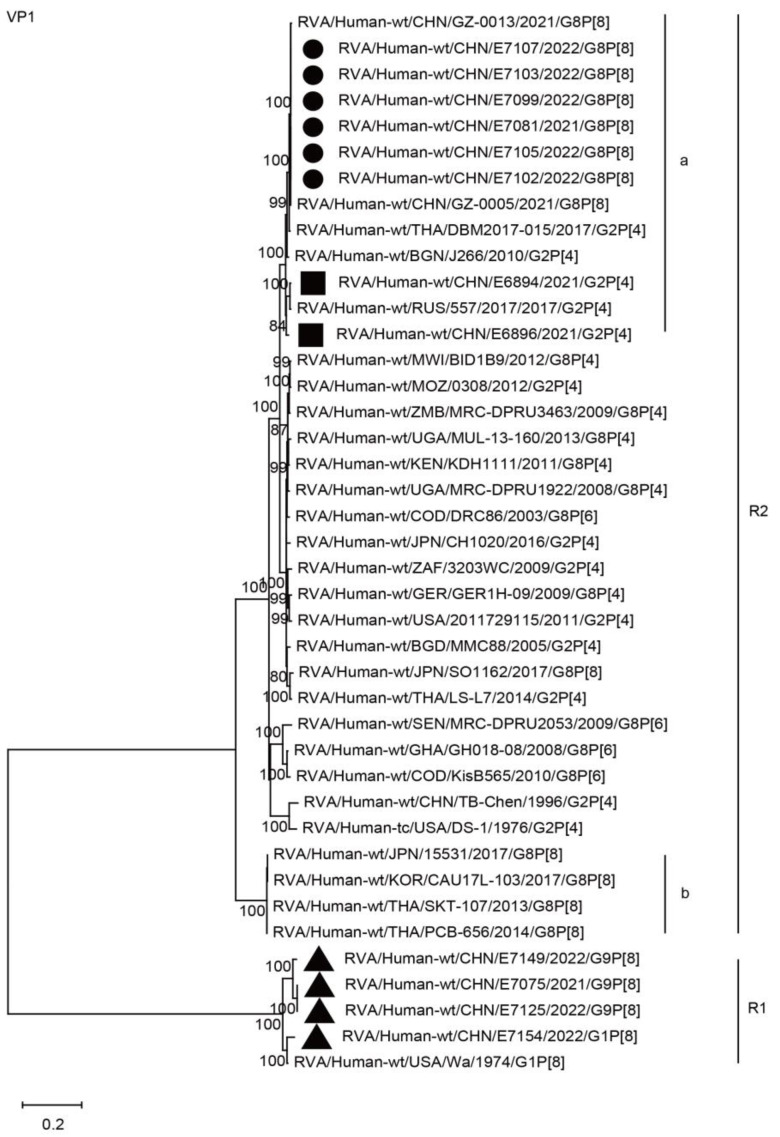
Phylogenetic dendrogram based on complete coding regions of the VP1 genes of representative RVAs. The best nucleotide substitution model was GTR + G + I. Bootstrap values below 80% are not shown. The G8P[8] strains are highlighted with a filled circle. The contemporaneous G2 and G1/G9 strains are highlighted with filled squares and triangles, respectively. “a” and “b” represent lineages within R2 (G8P[8] strains, lineage “a”).

The VP1 genes of all six strains in the present study were grouped into the R2 genotype. These VP1 genes clustered closely with those of two G8P[8] strains from Guangzhou City in China, the simultaneous circulating G2P[4] strains (E6896 and E6894) in Wuhan, and the G2P[4] strains in neighboring countries, with nucleotide identities of 97.0–99.1% (Figure 4, R2a), while remaining distant from the G8P[8] strains (15531, CAU17L-103, SKT-107, and PCB656) of neighboring countries, with nucleotide identities of 85.9–86.1% (Figure 4, R2b).

**Figure 5:**
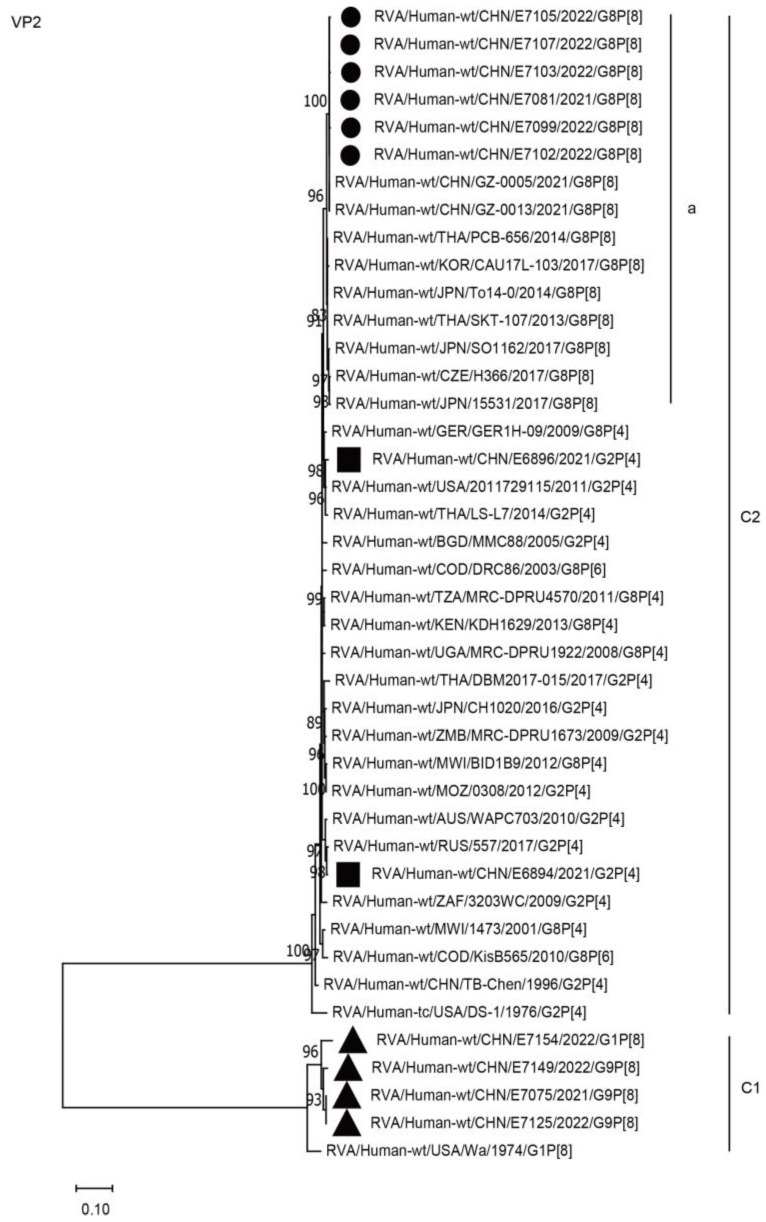
Phylogenetic dendrogram based on complete coding regions of the VP2 genes of representative RVAs. The best nucleotide substitution model was TN93 + G + I. Bootstrap values below 80% are not shown. The G8P[8] strains are highlighted with a filled circle. The contemporaneous G2 and G1/G9 strains are highlighted with filled squares and triangles, respectively. “a” represents a lineage of G8P[8] within C2.

**Figure 6:**
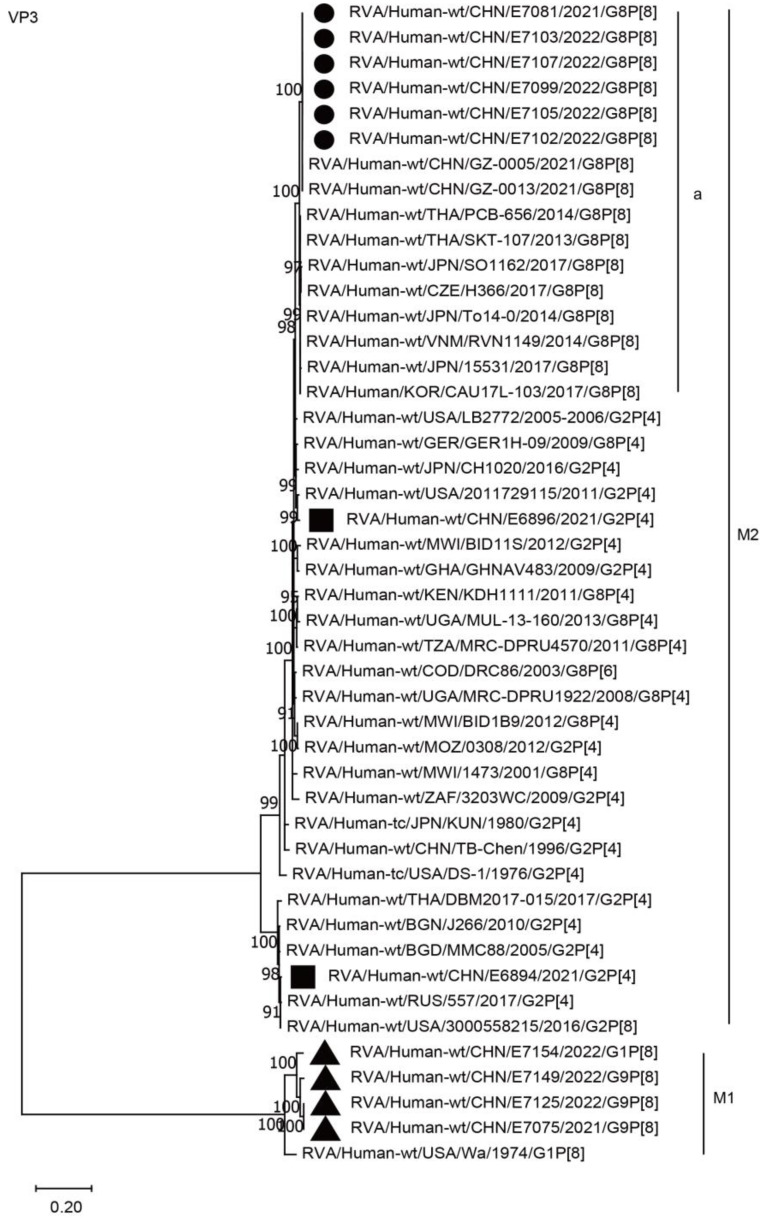
Phylogenetic dendrogram based on complete coding regions of the VP3 genes of representative RVAs. The best nucleotide substitution model was GTR + G + I. Bootstrap values below 80% are not shown. The G8P[8] strains are highlighted with a filled circle. The contemporaneous G2 and G1/G9 strains are highlighted with filled squares and triangles, respectively. “a” represents a lineage of G8P[8] within M2.

The VP2 and VP3 genes of all six G8P[8] strains in the present study were assigned to the C2 and M2 genotypes, respectively, and clustered closely with those of the G8P[8] strains from Guangzhou City in China (GZ-0005 and 0013), neighboring countries (PCB-656, CAU17L-103, TO14-0, and RVN1149), and Europe (H366), with high nucleotide identities of 97.4–99.8% (Figure 5, C2a; Figure 6, M2a; Table 2). The VP2 and VP3 genes of the six strains mentioned above were distant from the simultaneous co-circulating Chinese G2P[4] strains E6894 and E6896 detected in Wuhan, with nucleotide identities of 87.1–97.5% (Figure 5, C2, Figure 6, M2, Table 2).

#### 2.3.2. Nonstructural Protein Genes

**Figure 7:**
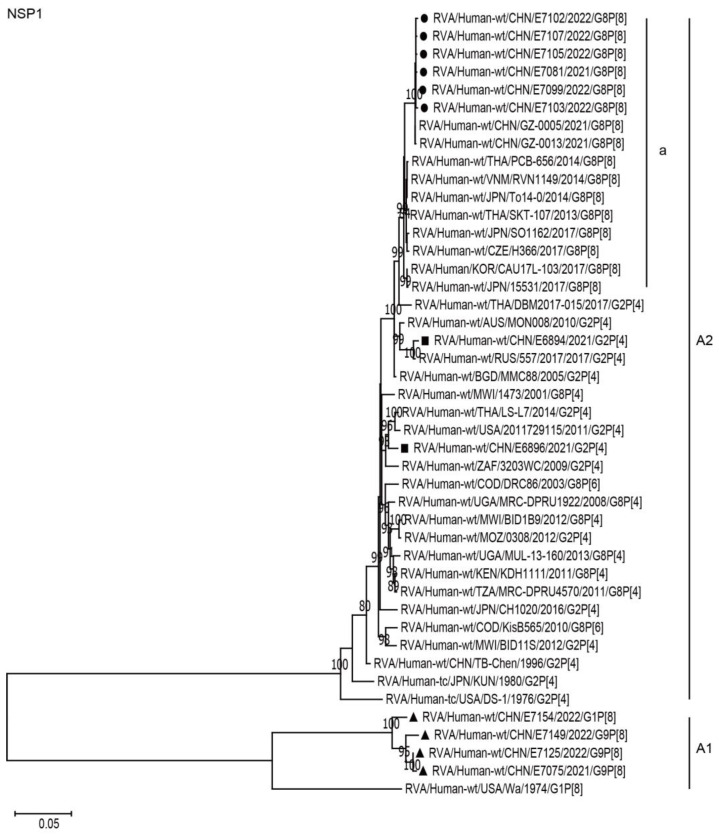
Phylogenetic dendrogram based on complete coding regions of the NSP1 genes of representative RVAs. The best nucleotide substitution model was T92 + G. Bootstrap values below 80% are not shown. The G8P[8] strains are highlighted with a filled circle. The contemporaneous G2 and G1/G9 strains are highlighted with filled squares and triangles, respectively. “a” represents a lineage of G8P[8] within A2.

**Figure 8:**
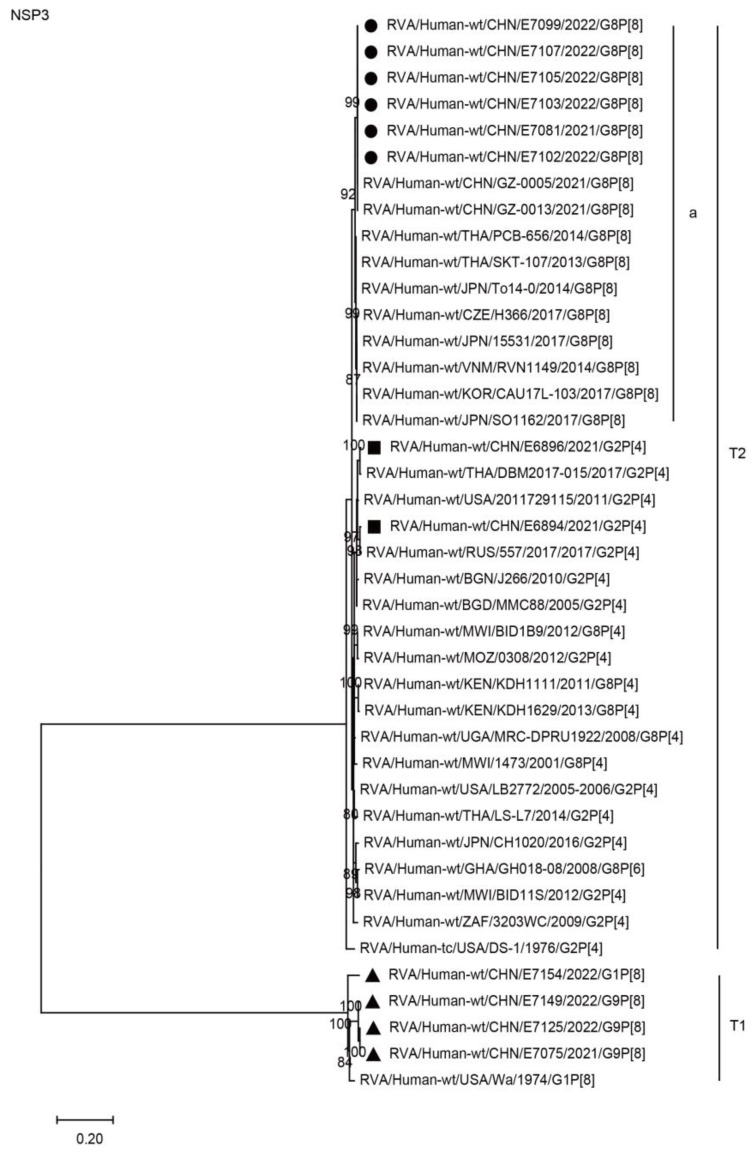
Phylogenetic dendrogram based on complete coding regions of the NSP3 genes of representative RVAs. The best nucleotide substitution model was T92 + G + I. Bootstrap values below 80% are not shown. The G8P[8] strains are highlighted with a filled circle. The contemporaneous G2 and G1/G9 strains are highlighted with filled squares and triangles, respectively. “a” represents a lineage of G8P[8] within T2.

**Figure 9:**
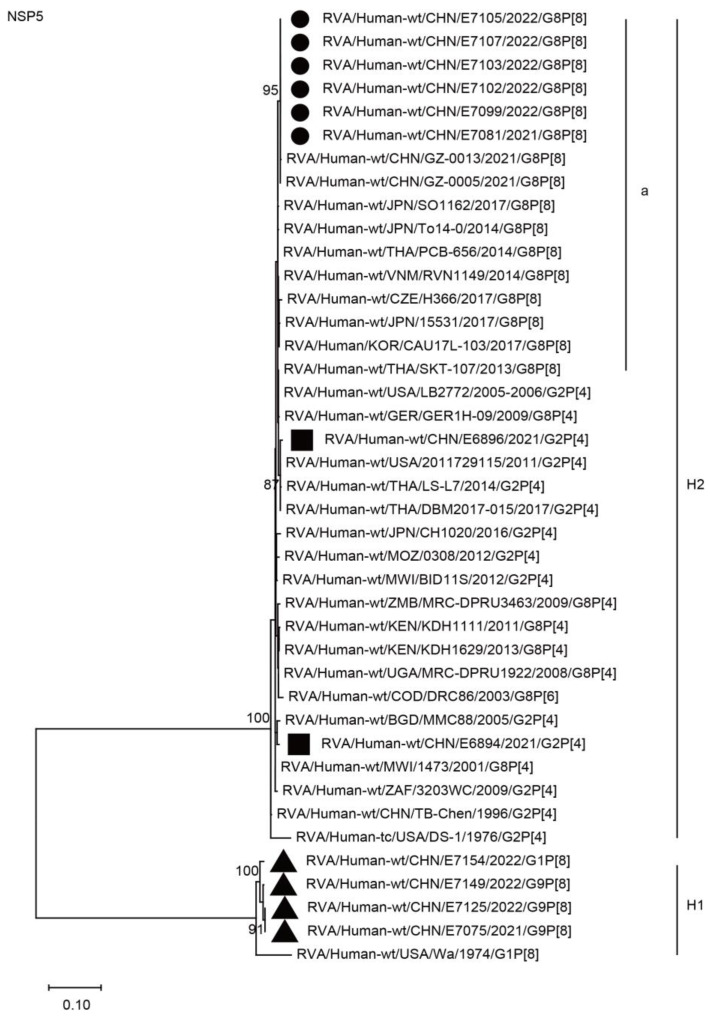
Phylogenetic dendrogram based on complete coding regions of the NSP5 genes of representative RVAs. The best nucleotide substitution model was T92 + I. Bootstrap values below 80% are not shown. The G8P[8] strains are highlighted with a filled circle. The contemporaneous G2 and G1/G9 strains are highlighted with filled squares and triangles, respectively. “a” represents a lineage of G8P[8] within H2.

The NSP1, NSP3, and NSP5 genes of the six G8P[8] strains were assigned to the A2, T2, and H2 genotypes, respectively. The NSP1, NSP3, and NSP5 genes of these G8P[8] strains clustered with those of two G8P[8] strains from Guangzhou City in China (GZ-0005 and 0013), showing high nucleotide identities (99.8–100%), and clustered together with the G8P[8] strains from neighboring countries (PCB-656, SO1162, SKT-107, 15531, CAU17L-103, TO14-0, and RVN1149) and Europe (H366), with high nucleotide identities of 98.2–99.5% (Figure 7, A2a; Figure 8, T2a; Figure 9, H2a; Table 2). The NSP1, NSP3, and NSP5 genes of the six strains mentioned above were distant from the simultaneously co-circulating G2P[4] strains E6894 and E6896 detected in Wuhan (Figure 7, Figure 8, Figure 9, and Table 2).

**Figure 10:**
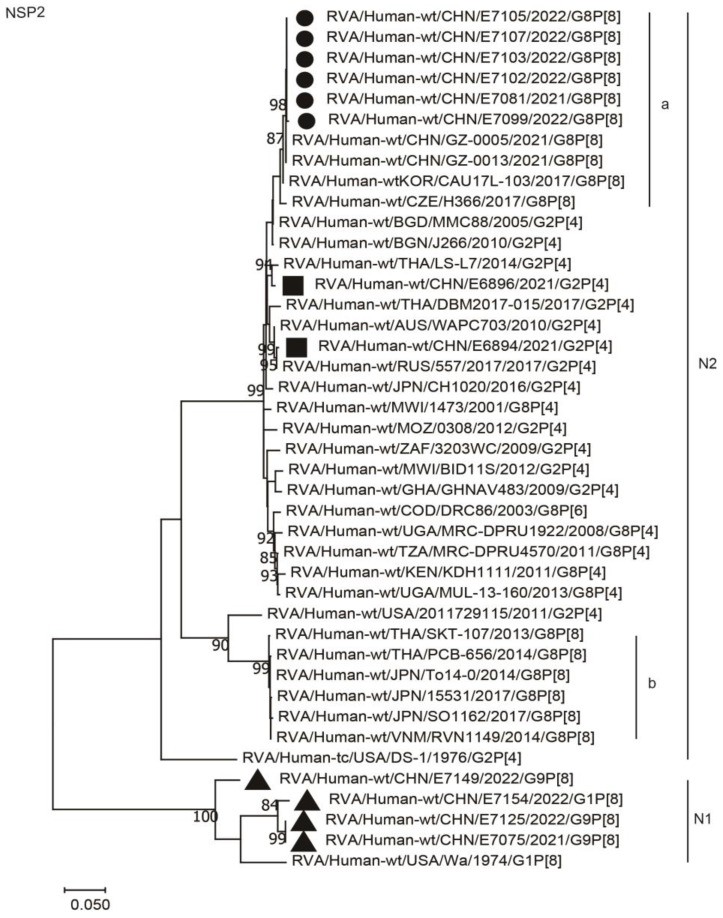
Phylogenetic dendrogram based on complete coding regions of the NSP2 genes of representative RVAs. The best nucleotide substitution model was T92 + G. Bootstrap values below 80% are not shown. The G8P[8] strains are highlighted with a filled circle. The contemporaneous G2 and G1/G9 strains are highlighted with filled squares and triangles, respectively. “a” and “b” represent lineages within N2 (G8P[8] strains, lineage “a”).

The NSP2 genes of the six G8P[8] strains were assigned to the N2 genotype. These NSP2 genes were distant from those of the co-circulating G2P[4] strains from Wuhan, showing nucleotide identities of 96.5%, and were more distant from some G8P[8] strains of neighboring countries (PCB-656, SO1162, SKT-107, 15531, TO14-0, and RVN1149), with lower nucleotide identities of 87.0–87.5% (Figure 10, Table 2). They clustered closely with two G8P[8] strains from Guangzhou City in the south of China (GZ-0005 and GZ0013), showing high nucleotide identities (99.9%), and were close to the G8P[8] strains from South Korea (CAU17L-103) and the Czech Republic (H366), with nucleotide identities of 99.5% and 98.3, respectively.

**Figure 11:**
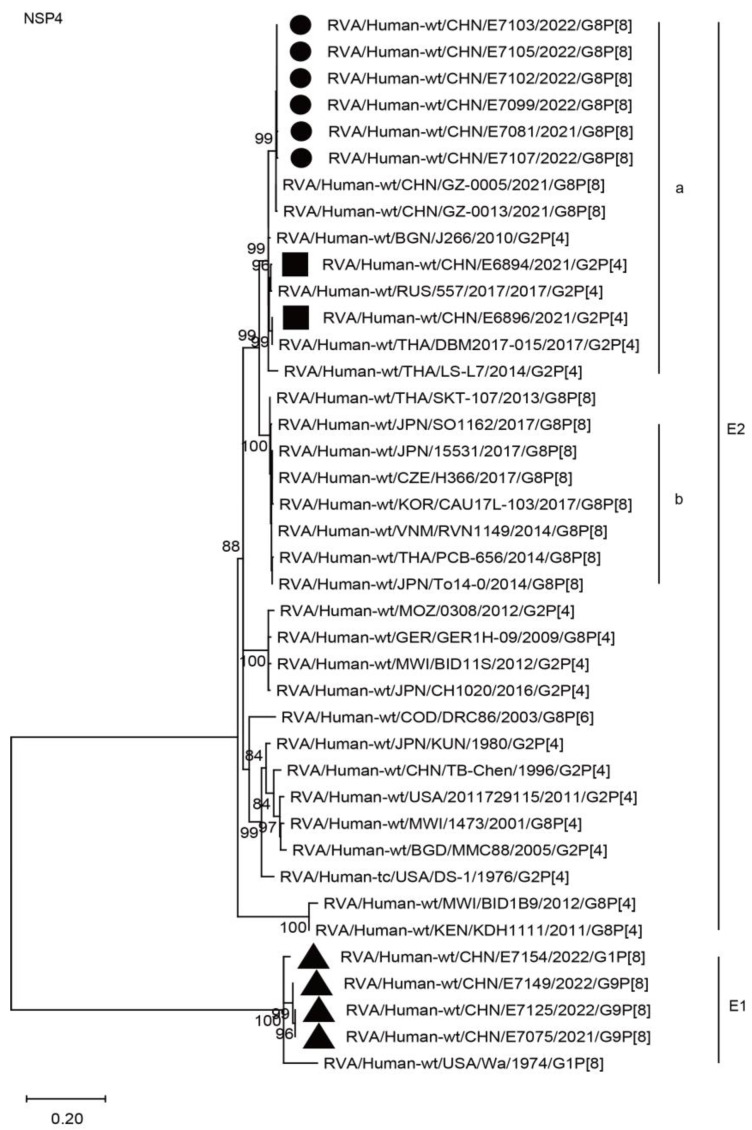
Phylogenetic dendrogram based on complete coding regions of the NSP4 genes of representative RVAs. The best nucleotide substitution model was T92 + I. Bootstrap values below 80% are not shown. The G8P[8] strains are highlighted with a filled circle. The contemporaneous G2 and G1/G9 strains are highlighted with filled squares and triangles, respectively. “a” and “b” represent lineages within E2 (G8P[8] strains, lineage “a”).

The NSP4 genes of the six G8 strains were assigned to the E2 genotype. They are closely related to two G8P[8] strains from Guangzhou City in the south of China, showing nucleotide identities of 99.4% and 99.6%, and they clustered together with the simultaneous G2P[4] strains (E6896 and E6894) in Wuhan and those of the G2P[4] strains in neighboring countries, while remaining distant from the G8P[8] strains (15531, CAU17L-103, SKT-107, and PCB656) detected in neighboring countries, showing nucleotide identities of 93.2–93.8% (Figure 11, H2a).

## 3. Discussion

Due to the COVID-19 pandemic, the lockdown from 23 January 2020 through 8 April 2020, and the subsequent zero-COVID-19 strategy, the collection of specimens was affected. The number of specimens decreased compared to a previous surveillance in Wuhan. In the previous surveillance of rotaviruses in Wuhan from 2011 through 2019, a total of 6733 fecal specimens from 4409 children (under 15 years old) and 2324 adults (15–96 years old) were collected. RVAs were detected in 25.5% (1125/4409) and 12.3% (285/2324) of the specimens from children and adults, respectively [27]. The average annual sample size from June 2011 through May 2019 was similar to that from phase 1 (pre-COVID-19 period). During phase 2 (outbreak and lockdown period) and phase 3 (zero-COVID-19 period), the average annual sample sizes were sharply reduced to 10.9% (60/551) and 36.3% (200/551) in children and 4.1% (12/291) and 10.7% (31/291) in adults, respectively.

Compared to the previous surveillance of rotaviruses in Wuhan from 2011 through 2019, the detection rate of RVAs in both children (χ^2^ = 15.01, *p* < 0.01) and adults (χ^2^ = 15.66, *p* < 0.01) declined in the present study. The public health measures taken during the COVID-19 era had a significant impact on people’s lives. Restrictions on the mobility of people and enhanced hand hygiene measures were deduced to be responsible for the lower incidence of RVAs both in children (19.40%) and adults (3.51%) that was observed in Wuhan with the background of the zero-COVID-19 strategy. Considering that the highest incidence was in the 13–36-month age group, the early introduction of routine rotavirus vaccinations to children before the kindergarten period is desired.

The Lanzhou lamb rotavirus (LLR) vaccine (G10P[15]) was licensed in China in 2000. It was the only rotavirus vaccine available before 12 April 2018, when RotaTeq (G1–G4, G6 P[8], P[5]) was introduced to mainland China. The LLR has been available in Wuhan since 2005. Because rotavirus vaccines are not included in the National Expanded Program of Immunization, the coverage is relatively low [28]. As discussed in the previous study, the influence of the LLR on the prevalence and genotypes of RVAs in Wuhan was limited [27]. A total of 185,507 doses of the LLR and 228,466 doses of RotaTeq were used from June 2019 through May 2022, which means about 61,835 and 76,155 infants were vaccinated with the LLR and RotaTeq, respectively. It was deduced that the use of rotavirus vaccines also contributed to the decline of the detection rates of RVAs.

G1/G3/G9-P[8] and G2P[4] were most commonly reported in China from 1994 to 2013 [29]. From 2000 through 2019, G1P[8], G3P[8], and G9P[8] were the predominant genotypes of RVAs that appeared in turn in Wuhan [24,25,26,27]. Compared with the previous surveillance, G9P[8] was still predominant, although the proportion declined. It is worth noting that G8 emerged in the 2021–2022 epidemic season in Wuhan and became a dominant strain. Although G8P[8] strains were detected in early 2021 in the coastal southern city of Guangzhou in China [9,10], our present study is the first report of the G8P[8] rotavirus that emerged in central mainland China.

The G8 RVA is a common genotype in cattle rotaviruses, especially in Africa [30]. Cattle and swine RVA strains may be able to infect other species through interspecies transmission [13]. Human G8 rotaviruses may be derived from a zoonotic infection, or alternatively, from a live rotavirus vaccine of bovine origin [16]. Human G8 has spread throughout Africa since 1990s, and it shifted from P[6] to P[4] and P[8] from 1997 to 2014 [31,32,33,34,35,36,37] (Table 2). G8P[8] human rotaviruses in Africa possessed the DS-1-like genetic background detected in Congo in 2003 [35]. Then, the DS-1-like G8P[8] rotavirus spread and circulated in Asian countries from 2010 through 2019 [18,19,20,21,22,23], and was subsequently reported in the Czech Republic and China [9,38].

Phylogenetic analysis revealed that the VP7, VP4, VP2, VP3, NSP1, NSP2, NSP3, and NSP5 genes of these G8P[8] strains clustered closely with those of the G8P[8] strains in Asia and were distant from those of the P[8] and G2P[4] strains simultaneously detected in Wuhan, while the VP1, VP6, and NSP4 genes were closely related to the typical G2P[4] rotavirus, including the G2P[4] strains detected in Wuhan. The present study suggests that the G8P[8] rotavirus emerging in China was generated by multiple reassortment events between the locally co-circulating G2P[4] rotavirus and the neighboring DS-1-like G8P[8] strains represented by CAU17L-103. The Chinese G8P[8] rotavirus carries the VP6, VP1, and NSP4 genes derived from the local G2P[4] rotavirus in the backbone of the neighboring DS-1-like G8P[8] strains. According to the emerging time of G8[8] strains in Guangzhou and Wuhan, the high nucleotide identity among these strains, and their closeness in phylogenetic trees, it was deduced that the G8P[8] strains in Guangzhou and Wuhan might have originated from the same rotavirus strain. The DS-1-like G8P[8] rotavirus might have emerged in coastal cities and then spread to the inland. These findings highlight the contribution of reassortment and interspecies transmission events to the high rotavirus diversity in mainland China.

Before the present study, the genomes of two G8P[8] strains from Guangzhou, China were analyzed [9]. However, due to a lack of data on the local co-circulating Wa and DS-1 genogroup strains, the reassortment between local G2P[4] strains and Asian DS-1-like G8P[8] strains could not be revealed.

With the accumulation of whole-genome data for rotaviruses in humans and animals, we can further understand the evolution of the DS-1-like G8P[8] rotavirus and the spread of G8 rotaviruses in humans. The G6, G8, G10, and G15 genotypes are mostly combined with the typical bovine DS-1-like genomic constellation [39,40,41]. The DS-1-like genomic constellation G10/G6-P[11]/P[5]-I2-R2-C2-M2-A3/A11/A13-N2-T6-E2-H3 is the most prevalent in cattle. The genotypes N2-T6-E2-H3 are constant in the genomic constellations of bovine rotaviruses. It was deduced that the evolution process of the DS-1-like G8P[8] rotavirus might have been accompanied by interspecies transmission and multiple genetic reassortment events between bovine/human and human RVAs. Finally, the VP7 gene from bovine strains and the VP4 gene from human strains are present on the backbone of the human DS-1-like rotavirus.

The present study is helpful for understanding the evolutionary dynamics of emerging DS-1-like G8P[8] strains, and it provides new evidence that the DS-1-like G8P[8] rotavirus adapted to become a predominant human rotavirus strain and an important pathogen in the world [34]. Neither LLR nor RotaTeq can cover the emerging G8P[8] rotavirus. Monitoring the emerging genotypes in humans will assist scientists in developing or importing new vaccines and adjust the components of existing vaccines to improve their protective effect. The continuous surveillance of rotaviruses in both animals and humans is necessary.

## 4. Materials and Methods

### 4.1. Specimens

A hospital-based surveillance of sporadic diarrhea was conducted in Wuhan, China. The definition of diarrhea is loose/watery stools that occur three or more times within 24 h. The stool specimens were collected from inpatients and outpatients in four hospitals (Wuhan Commercial Staff Hospital, Wuhan Sixth Hospital, Wuhan Eleventh Hospital, and Wuhan Children’s Hospital). All the specimens were stored at −80 °C. The concise epidemiological and clinical data were collected with a sampling form.

### 4.2. Detection of Rotavirus

Viral dsRNA was extracted from 200 microliters of a 10% stool suspension with a nucleic acid extraction kit by using the automatic nucleic acid extraction system NP968S (Suzhou Tianlong Science and Technology Co., Ltd., Suzhou, China) according to the manufacturer’s instructions. Using real-time reverse transcription–polymerase chain reaction (RT-PCR) with a rotavirus kit (Zhijiang Science and Technology Co., Ltd., Shanghai, China), RVAs, RVBs, and RVCs were detected.

### 4.3. Genotyping of RVAs

The nearly full-length sequence of the VP7 gene and the partial VP4 gene were amplified using PrimeScript™ One Step RT-PCR Kit Ver.2 (Takara Biomedical Technology (Beijing) Co., Ltd. Beijing, China) according to the manufacturer’s instructions. The nucleotide sequences of the primers are listed in Table S1. The genotypes of the VP7 and VP4 genes were determined by analyzing the sequences of the PCR products [42,43,44]. The PCR products were subjected to direct sequencing by the Sanger method at the Sangon Biotech (Shanghai) Co., Ltd. branch of Wuhan (Wuhan, China). The genotypes of RVAs were preliminarily assigned by the basic local alignment search tool (BLAST) and then confirmed by an analysis of phylogenetic trees.

### 4.4. Whole-Genome Sequencing

The complete eleven segmented genes of the whole genome were amplified by PrimeScript™ One Step RT-PCR Kit Ver.2 (Takara Biomedical Technology (Beijing) Co., Ltd. Beijing, China) according to the manufacturer’s instructions. The nucleotide sequences of the primers are listed in Table S1 and include those reported in the previous studies [42,43,44,45,46]. Of these, the nearly full-length sequences of the VP4, VP6, VP1–3, and NSP1 genes were obtained by segmented RT-PCR and sequencing. The PCR products were subjected to direct sequencing by the Sanger method at the Sangon Biotech (Shanghai) Co., Ltd. branch of Wuhan (Wuhan, China).

### 4.5. Phylogenetic Analysis

The nucleotide sequence of each segment was assembled and edited using the DNAMAN v10 software. The sequence identities were analyzed by the Lasergene bio-information v7 software (DNASTAR, Inc, Madison, WI, USA). Multiple alignments of the sequences were performed using MAFFT v7 (Kazutaka Katoh, Osaka, Japan) [47]. A phylogenetic analysis was conducted together with reference strain sequences obtained from the National Center for Biotechnology Information (NCBI) database by the MEGA program, version X [48]. The evolutionary history was inferred by using the maximum likelihood method, with 1000 bootstrap replicates, based on the best nucleotide substitution model with the lowest Bayesian information criterion (BIC) score in MEGA X [49]. The initial tree for the heuristic search was obtained automatically by applying neighbor-joining and BioNJ algorithms to a matrix of pairwise distances estimated using the maximum composite likelihood (MCL) approach, and then selecting the topology with a superior log likelihood value. The tree was drawn to scale, with branch lengths represented by the number of substitutions per site.

### 4.6. Statistical Analysis

Statistical analyses were performed using the Statistical Package for Social Science (SPSS, version 24, SPSS Inc., Chicago, IL, USA) software. Testing for the statistical significance was performed using the chi-squared test. A *p*-value less than 0.05 was considered significant.

### 4.7. Accession Numbers of Nucleotide Sequences in Genbank

The genome sequences of the strains, including RVA/Human-wt/CHN/E7081/2021/G8P[8], RVA/Human-wt/CHN/E7099/2022/G8P[8], RVA/Human-wt/CHN/E7102/2022/G8P[8], RVA/Human-wt/CHN/E7103/2022/G8P[8], RVA/Human-wt/CHN/E7105/2022/G8P[8], RVA/Human-wt/CHN/E7107/2022/G8P[8], RVA/Human-wt/CHN/E6894/2021/G2P[4], RVA/Human-wt/CHN/E6896/2021/G2P[4], RVA/Human-wt/CHN/E7154/2022/G1P[8], RVA/Human-wt/CHN/E7075/2021/G9P[8], RVA/Human-wt/CHN/E7125/2022/G9P[8], and RVA/Human-wt/CHN/E7149/2022/G9P[8], were deposited in the GenBank database under the accession numbers OP850379-387, OP850392-400, OP850405-413, OP450418-426, OP450431-439, OP450444-452, OP450457-465, OP450470-478, OP450483-491, OP450496-504, and OP450509-517.

## Figures and Tables

**Table 1:** Frequency of G and P genotypes of rotaviruses detected in Wuhan from June 2019 through May 2022.

G-Type/P-Type	June 2019–May 2020		June 2020–May 2021		June 2021–May 2022		Total	
	Child	Adult	Child	Adult	Child	Adult	Child	Adult
G1/P[8]	0	0	1	0	3	0	4	0
G2/P[4]	0	0	5	0	0	0	5	0
G3/P[8]	15	0	7	0	0	0	22	0
G8/P[8]	0	0	0	0	22	0	22	0
G9/P[8]	42	4	34	0	13	4	89	8
Total	57	4	47	0	38	4	142	8

**Table 2:** Comparison of G8P[8] rotaviruses in Wuhan with other representative rotaviruses of Wa-like and DS-1-like genogroups.

Strain	Genotypes of Viral Protein Genes and Nucleotide Sequence Identities (%) to E7081																					
	VP7		VP[4]		VP6		VP1		VP2		VP3		NSP1		NSP2		NSP3		NSP[4]		NSP5	
**RVA/Human-wt/CHN/E7081/2021/G8P[8]**	**G8**	—	**P[8]**	—	**I2**	—	**R2**	—	**C2**	—	**M2**	—	**A2**	—	**N2**	—	**T2**	—	**E2**	—	**H2**	—
**RVA/Human-wt/CHN/E7099/2022/G8P[8]**	**G8**	100.0	**P[8]**	100.0	**I2**	100.0	**R2**	99.9	**C2**	99.8	**M2**	100.0	**A2**	99.9	**N2**	99.8	**T2**	99.9	**E2**	99.8	**H2**	100.0
**RVA/Human-wt/CHN/E7102/2022/G8P[8]**	**G8**	99.9	**P[8]**	100.0	**I2**	99.9	**R2**	99.9	**C2**	99.9	**M2**	99.9	**A2**	99.8	**N2**	100.0	**T2**	99.9	**E2**	99.8	**H2**	100.0
**RVA/Human-wt/CHN/E7103/2022/G8P[8]**	**G8**	99.9	**P[8]**	100.0	**I2**	99.9	**R2**	99.9	**C2**	99.7	**M2**	99.9	**A2**	99.8	**N2**	100.0	**T2**	100.0	**E2**	99.8	**H2**	100.0
**RVA/Human-wt/CHN/E7105/2022/G8P[8]**	**G8**	99.9	**P[8]**	99.8	**I2**	99.9	**R2**	99.9	**C2**	99.8	**M2**	100.0	**A2**	99.9	**N2**	100.0	**T2**	100.0	**E2**	99.8	**H2**	100.0
**RVA/Human-wt/CHN/E7107/2022/G8P[8]**	**G8**	100.0	**P[8]**	99.9	**I2**	99.9	**R2**	99.9	**C2**	99.9	**M2**	99.9	**A2**	99.9	**N2**	100.0	**T2**	99.9	**E2**	99.6	**H2**	100.0
**RVA/Human-wt/CHN/E6894/2021/G2P[4]**	G2	73.1	P[4]	87.0	**I2**	96.4	**R2**	97.0	**C2**	96.7	**M2**	87.1	**A2**	96.5	**N2**	96.5	**T2**	96.4	**E2**	97.5	**H2**	98.4
**RVA/Human-wt/CHN/E6896/2021/G2P[4]**	G2	73.3	P[4]	86.6	**I2**	97.7	**R2**	97.6	**C2**	97.5	**M2**	96.2	**A2**	95.7	**N2**	96.5	**T2**	95.8	**E2**	97.0	**H2**	98.7
**RVA/Human-wt/CHN/E7154/2022/G1P[8]**	G1	73.4	**P[8]**	95.7	I1	79.5	R1	79.3	C1	79.1	M1	77.4	A1	75.0	N1	83.4	T1	79.6	E1	80.7	H1	83.4
**RVA/Human-wt/CHN/E7075/2021/G9P[8]**	G9	76.2	**P[8]**	97.2	I1	80.1	R1	80.1	C1	79.4	M1	77.1	A1	74.9	N1	82.8	T1	79.3	E1	80.5	H1	83.9
**RVA/Human-wt/CHN/E7125/2022/G9P[8]**	G9	76.2	**P[8]**	97.2	I1	80.1	R1	80.1	C1	79.4	M1	77.1	A1	75.1	N1	82.8	T1	79.3	E1	80.5	H1	83.9
**RVA/Human-wt/CHN/E7149/2022/G9P[8]**	G9	76.1	**P[8]**	97.2	I1	79.9	R1	80.0	C1	79.1	M1	77.2	A1	75.2	N1	83.3	T1	79.2	E1	80.3	H1	83.6
RVA/Human-wt/CHN/GZ-0005/2021/G8P[8]	G8	99.9	P[8]	99.7	I2	99.7	R2	99.8	C2	99.7	M2	99.7	A2	99.9	N2	99.9	T2	99.9	E2	99.6	H2	100.0
RVA/Human-wt/CHN/GZ-0013/2021/G8P[8]	G8	99.7	P[8]	99.8	I2	99.7	R2	99.8	C2	99.8	M2	99.7	A2	99.8	N2	99.9	T2	99.9	E2	99.4	H2	99.8
RVA/Human-wt/KOR/CAU17L-103/2017/G8P[8]	G8	99.4	P[8]	98.3	I2	96.4	R2	86.1	C2	98.7	M2	98.3	A2	98.6	N2	99.5	T2	98.4	E2	93.4	H2	98.2
RVA/Human-wt/JPN/15531/2017/G8P[8]	G8	99.2	P[8]	98.2	I2	96.4	R2	86.1	C2	98.4	M2	98.2	A2	98.6	N2	87.3	T2	98.6	E2	93.2	H2	99.0
RVA/Human-wt/JPN/SO1162/2017/G8P[8]	G8	99.3	P[8]	98.2	I2	96.5	R2	93.8	C2	98.5	M2	98.4	A2	98.6	N2	87.0	T2	98.4	E2	93.8	H2	99.3
RVA/Human-wt/CZE/H366/2017/G8P[8]	G8	99.3	P[8]	98.1	I2	96.3	R2	86.1	C2	98.3	M2	98.4	A2	98.6	N2	98.3	T2	98.3	E2	93.2	H2	98.7
RVA/Human-wt/THA/PCB-656/2014/G8P[8]	G8	99.4	P[8]	98.2	I2	96.6	R2	86.0	C2	97.4	M2	98.6	A2	98.7	N2	87.2	T2	98.8	E2	93.2	H2	99.5
RVA/Human-wt/JPN/To14-0/2014/G8P[8]	G8	98.2	P[8]	98.3	I2	96.6	R2	85.9	C2	99.0	M2	98.6	A2	98.8	N2	87.3	T2	98.7	E2	93.4	H2	99.3
RVA/Human-wt/VNM/RVN1149/2014/G8P[8]	G8	99.4	P[8]	98.3	I2	96.6	R2	86.0	C2	98.7	M2	98.5	A2	98.8	N2	87.2	T2	98.6	E2	93.4	H2	99.3
RVA/Human-wt/THA/SKT-107/2013/G8P[8]	G8	99.2	P[8]	98.4	I2	91.3	R2	86.1	C2	99.0	M2	98.6	A2	98.8	N2	87.5	T2	98.8	E2	93.8	H2	99.3
RVA/Human-wt/COD/DRC86/2003/G8P[6]	G8	86.2	P[6]	75.3	I2	96.2	R2	94.3	C2	97.0	M2	96.5	A2	95.9	N2	95.6	T2	96.9	E2	89.2	H2	97.9
RVA/Human-wt/KEN/KDH1629/2013/G8P[4]	G8	86.1	P[4]	87.4	I2	95.7	R2	94.3	C2	97.4	M2	96.0	A2	96.0	N2	95.3	T2	96.4	E2	85.2	H2	98.5
RVA/Human-wt/MWI/1473/2001/G8P[4]	G8	86.9	P[4]	87.0	I2	97.8	R2	86.3	C2	96.8	M2	95.6	A2	96.2	N2	96.1	T2	97.0	E2	88.8	H2	99.0
RVA/Human-wt/THA/DBM2017-015/2017/G2P[4]	G2	73.2	P[4]	86.8	I2	97.8	R2	99.1	C2	96.2	M2	87.2	A2	97.6	N2	96.3	T2	95.7	E2	97.0	H2	99.0
RVA/Human-tc/USA/DS-1/1976/G2P[4]	G2	72.6	P[4]	86.9	I2	87.4	R2	90.2	C2	93.7	M2	92.6	A2	92.6	N2	86.1	T2	94.5	E2	89.8	H2	95.7
RVA/Human-wt/USA/Wa/1974/G1P[8]	G1	73.2	P[8]	90.1	I1	79.6	R1	79.7	C1	80.0	M1	77.0	A1	75.4	N1	82.0	T1	79.6	E1	80.7	H1	83.7

All strains detected in the present study are indicated by a yellow background and bold, including their genotypes same as the emerging DS-1-like G8P[8] rotaviruses.

## Data Availability

The data will be provided upon request.

## Supplementary Materials

The supporting information can be downloaded at: https://www.mdpi.com/article/10.3390/ijms241512189/s1.
